# A systematic review and meta-analysis of the efficacy of exercise therapy for the rehabilitation of rotator cuff-related shoulder pain: a subgroup analysis of racquet sport athletes

**DOI:** 10.3389/fbioe.2026.1840050

**Published:** 2026-06-04

**Authors:** Jingyi Wu, Yikun Zheng, Wenzhi Cai, Daisuke Takeshita, Ping Yeap Loh, Guopeng Hu, Yuqi He

**Affiliations:** 1 College of Physical Education, Huaqiao University, Quanzhou, China; 2 Department of Life Sciences, Graduate School of Arts and Sciences, The University of Tokyo, Tokyo, Japan; 3 Department of Life Design and Science, Faculty of Design, Kyushu University, Fukuoka, Japan

**Keywords:** exercise therapy, meta-analysis, racquet sport athletes, rotator cuff-related shoulder pain, sports rehabilitation, therapeutic efficacy

## Abstract

**Objective:**

Rotator Cuff Related Shoulder Pain (RCRSP) is a common musculoskeletal disorder in both clinical and athletic populations, with a particularly high prevalence among racquet sport athletes. This study aimed to systematically evaluate the overall effectiveness of exercise therapy in the rehabilitation of RCRSP and to further explore its therapeutic effects among racquet sport athletes, providing evidence-based guidance for clinical practice and individualized rehabilitation programs.

**Methods:**

Following the PRISMA guidelines, a systematic search was conducted across PubMed, Web of Science, Cochrane Library, Embase, CNKI, VIP, and Wanfang databases. Randomized controlled trials (RCTs) investigating the effects of exercise therapy on RCRSP were included. Meta-analysis was performed using Stata 18 software with a random-effects model for pooled analysis. Subgroup and sensitivity analyses were conducted to explore potential influencing factors.

**Results:**

Eight RCTs were included. The pooled analysis showed no evidence of a difference between exercise therapy and control for shoulder pain (SMD = 0.01, 95% CI −0.21 to 0.24; p = 0.93). Heterogeneity was considerable (I^2^ = 96.5%; Cochran’s Q p < 0.001). Sensitivity analysis indicated that the pooled effect size remained stable after removing individual studies (range: −1.24 to 1.13). Subgroup analysis revealed significant differences in treatment efficacy across different athlete populations (between-group difference test p < 0.001). Among racquet sport athletes, exercise therapy significantly reduced pain (SMD = −0.94, 95% CI: −1.38 to −0.50, p < 0.001). In the non-racket sports athletes subgroup, the pooled effect size was SMD = 0.35 (95% CI: 0.09 to 0.61, p < 0.001), with inconsistent effect direction. The overall pooled effect was non-significant (SMD = 0.01, 95% CI: −0.21–0.24). The funnel plot does not show clear symmetry, suggesting possible small-study effects or publication bias.

**Conclusion:**

This study suggested that exercise therapy may be associated with greater pain reduction among racquet sport athletes. Given the substantial heterogeneity, limited number of included studies, and potential publication bias, these findings should be interpreted with caution. Future high-quality randomized controlled trials are needed to confirm the effectiveness of exercise therapy in sport-specific populations.

## 1 Introduction

Rotator Cuff Related Shoulder Pain (RCRSP) is one of the most common shoulder conditions encountered in clinical practice, primarily characterized by shoulder joint pain, limited range of motion, and decreased function. This condition has a relatively high incidence in the general population, with a lifetime prevalence of approximately 70% (Struyf, 2024). It is particularly common among athletes, especially racquet sports players. Prolonged, repetitive upper limb extension and shoulder rotation movements significantly increase susceptibility to chronic overuse or degenerative damage of rotator cuff tissues (Baysal et al., 2005). RCRSP not only impacts athletic performance and competitive outcomes but may also cause long-term shoulder dysfunction, severely affecting quality of life in severe cases (Desmeules et al., 2025). Treatment approaches for RCRSP include medication, physical therapy, injection therapy, and surgery (Lafrance et al., 2024). Among these, exercise therapy—a non-invasive, cost-effective, and sustainable conservative treatment option—has garnered significant attention in recent years. Exercise therapy alleviates pain and restores function by improving shoulder joint range of motion, strengthening rotator cuff muscles, enhancing scapular stability, and promoting neuromuscular control (Wu et al., 2025). Research indicates that scientifically designed exercise interventions can effectively improve functional impairments in patients with RCRSP (Raulline Ullern et al., 2025). However, variations in intervention type, intensity, and implementation methods across existing studies result in inconsistent outcomes (Cavaggion et al., 2024). Some studies suggest that “exercise into pain” training yields superior rehabilitation outcomes, while others advocate for pain-free or low-intensity training as safer and more effective. Consequently, no unified consensus exists regarding the optimal form of exercise therapy intervention (Haidich, 2010).

With the advancement of evidence-based medicine, systematic reviews and meta-analyses have become crucial methods for integrating diverse research findings and elevating the level of evidence. While some domestic and international studies have reviewed the efficacy of exercise therapy for rotator cuff injuries or shoulder pain, most focus on the general population. Systematic analyses targeting specific athletic populations (e.g., racquet sports athletes) remain scarce (Guérineau et al., 2025). Furthermore, different exercise modalities, training intensities, and pain management strategies may exert varying effects on rehabilitation outcomes, necessitating further clarification through systematic integration. Racquet sport athletes represent a clinically relevant subgroup because their shoulder loading pattern differs from that of the general population (Garret et al., 2024). Sports such as tennis, badminton, table tennis, and squash require repeated overhead or high-velocity upper-limb movements, rapid acceleration and deceleration of the shoulder, and frequent internal–external rotation under sport-specific technical demands (Kovacs and Ellenbecker, 2011; Pardiwala et al., 2020). These repetitive mechanical loads may increase stress on the rotator cuff and surrounding shoulder stabilizers (Alrabaa et al., 2020). In addition, rehabilitation in this population is not limited to pain reduction alone, but also requires restoration of shoulder strength, dynamic stability, neuromuscular control, and sport-specific movement capacity for safe return to training and competition (Oak et al., 2022). Therefore, this study employs systematic review and meta-analysis methods to comprehensively evaluate the efficacy of exercise therapy in rehabilitating shoulder pain associated with rotator cuff injuries, with a subgroup analysis focusing on racquet sport athletes. Through quantitative synthesis and comparison of randomized controlled trials from domestic and international sources, this study aims to clarify the overall efficacy of exercise therapy and potential differences among various intervention approaches. It seeks to provide evidence-based guidance for the rehabilitation of RCRSP patients and offer reference for clinical interventions and individualized exercise prescription in sports-related shoulder pain.

## 2 Materials and methods

### 2.1 Literature search

This systematic review and meta-analysis was conducted in accordance with the PRISMA guidelines and was prospectively registered in PROSPERO (Registration ID: CRD420251167881) (Page et al., 2021). A comprehensive literature search was performed in PubMed, Web of Science, Cochrane Library, Embase, CNKI, VIP, and Wanfang databases from database inception to 15 October 2025.

The search strategy was developed around three main concepts: exercise therapy, rotator cuff-related shoulder pain, and sport or athlete populations. The English search terms included combinations of the following keywords and Medical Subject Headings where applicable: “exercise therapy,” “therapeutic exercise,” “physical therapy,” “rehabilitation exercise,” “rotator cuff,” “rotator cuff injury,” “rotator cuff-related shoulder pain,” “shoulder pain,” “subacromial pain syndrome,” “athlete,” “sport,” “racquet sport,” “racket sport,” “tennis,” “badminton,” “table tennis,” and “squash.” The Chinese search terms included equivalent terms related to exercise therapy, physical therapy, rotator cuff injury, shoulder pain, sports rehabilitation, athletes, and racquet sports. The detailed search strategy for each database is presented in Table 1.

**TABLE 1:** Search strategy and results table.

Database	Search strategy	Number of documents	Reasons for exclusion	Number of documents included
PubMed	(“exercise therapy” OR “physical therapy”) AND (“rotator cuff injury”) AND (“sports rehabilitation”) AND (“racquet sport athletes” OR “adults”) AND (“Meta-analysis”)	276	Duplicate literature, non-randomized controlled trials	4
Web of Science	(“exercise therapy” OR “physical therapy”) AND (“rotator cuff injury”) AND (“sports rehabilitation”) AND (“racquet sport athletes” OR “adults”) AND (“Meta-analysis”)	50	Systematic reviews and systematic evaluation articles	1
Cochrane Library	(“exercise therapy” OR “physical therapy”) AND (“rotator cuff injury”) AND (“sports rehabilitation”) AND (“racquet sport athletes” OR “adults”) AND (“Meta-analysis”)	7	No complete data	0
Embase	(“exercise therapy” OR “physical therapy”) AND (“rotator cuff injury”) AND (“sports rehabilitation”) AND (“racquet sport athletes” OR “adults”) AND (“Meta-analysis”)	13	Non-relevant literature	0
CNKI	(“Exercise therapy” OR “physical therapy”) AND (“rotator cuff injury”) AND (“sports rehabilitation”) AND (‘adults’ OR “racquet athletes”) AND (“meta-analysis”)	120	Duplicate literature, non-randomized controlled trials	3
VIP	(“Exercise therapy” OR “physical therapy”) AND (“rotator cuff injury”) AND (“sports rehabilitation”) AND (‘adults’ OR “racquet athletes”) AND (“meta-analysis”)	10	No complete data	0
Wanfang database	(“Exercise therapy” OR “physical therapy”) AND (“rotator cuff injury”) AND (“sports rehabilitation”) AND (‘adults’ OR “racquet athletes”) AND (“meta-analysis”)	4	Non-randomized controlled trials	0

No restriction was applied to publication date. Studies published in English or Chinese were considered eligible. The study type was restricted to randomized controlled trials. Systematic reviews, narrative reviews, conference abstracts, case reports, animal studies, and studies without extractable outcome data were excluded. Unpublished studies and grey literature were not included because the present review focused on peer-reviewed studies with sufficient methodological and outcome information. To improve the comprehensiveness of the search, the reference lists of the included studies and relevant reviews were also manually screened.

### 2.2 Literature screening

One researcher independently used Zotero software (version 6.0.30; Roy Rosenzweig Center for History and New Media, USA) to deduplicate and manage retrieved literature. Jingyi Wu and Yikun Zheng independently screened titles/abstracts and then full texts according to predefined inclusion and exclusion criteria (Table 2). Disagreements were first resolved through discussion; if consensus could not be reached, Wenzhi Cai acted as the arbitrator and made the final decision. Literature screening was completed on 25 October 2025.

**TABLE 2:** Inclusion and exclusion criteria table.

PICOS	Inclusion criteria	Exclusion criteria
Study population	The study subjects were patients with shoulder pain related to rotator cuff injuries, with a focus on racquet sports athletes (such as badminton, tennis, and table tennis players)	(1) Patients without rotator cuff injuries: Such as other shoulder joint conditions (acromioclavicular impingement, shoulder dislocation, etc.); (2) studies lacking an effective control group or with insufficient data; (3) interventions that were unclear or failed to detail the specific content of exercise therapy; (4) studies with insufficient methodological information for risk-of-bias assessment were excluded.
Intervention measures	Exercise therapy as the primary intervention, including core stability training and joint range-of-motion exercises	
Comparison method	Physical therapy, surgical treatment, or conventional treatment	
Outcome Indicator	The primary outcome measure is the pain VAS score, with secondary outcome measures including shoulder range of motion and constant-murley score	
Research design	Randomized controlled trial	

### 2.3 Inclusion and exclusion criteria

Inclusion and exclusion criteria were determined based on the PICOS framework. Studies were included if they met the following criteria: participants were diagnosed with rotator cuff-related shoulder pain or rotator cuff injury; the intervention included exercise therapy or rehabilitation exercise; the comparator was conventional treatment, physical therapy, usual care, or other non-exercise or alternative rehabilitation approaches; pain-related outcomes such as VAS were reported; and the study design was a randomized controlled trial. Studies were excluded if they were non-randomized studies, systematic reviews, narrative reviews, conference abstracts, case reports, animal studies, duplicate publications, or if the full text or necessary outcome data could not be obtained.

### 2.4 Data extraction and transformation

On 1 November 2025, Jingyi Wu and Yikun Zheng independently extracted data from all included studies using a standardized, piloted extraction form developed according to the PICOS framework. Extracted items included study authors, publication year, participant characteristics, intervention and comparator details, and outcome measures. When available, follow-up duration and post-intervention assessment time points were also extracted. However, because follow-up data were not consistently reported across the included studies, long-term effects were not pooled separately. After extraction, the two datasets were cross-checked; discrepancies were resolved by discussion and, if necessary, adjudicated by Wenzhi Cai. For outcome measures, this study primarily selected VAS scores, joint range of motion, and Constant-Murley scores. Since VAS scores were consistently used across all included studies, this study calculated outcomes based on VAS scores.

### 2.5 Bias risk and methodological quality

The risk of bias of the included randomized controlled trials was assessed using the Cochrane Risk of Bias two tool. Two reviewers independently evaluated each included study across the following domains: bias arising from the randomization process, bias due to deviations from the intended interventions, bias due to missing outcome data, bias in measurement of the outcome, and bias in selection of the reported result. Each domain was judged as “low risk of bias,” “some concerns,” or “high risk of bias,” and an overall risk-of-bias judgment was then assigned for each study. Any disagreements between the two reviewers were first resolved through discussion. If consensus could not be reached, a third reviewer acted as an arbitrator. This procedure was applied to improve the consistency and transparency of the assessment process. No study was excluded or statistically down-weighted solely on the basis of risk-of-bias judgment; instead, the risk-of-bias assessment was used to inform the interpretation of the findings and the certainty of evidence assessment.

### 2.6 Levels of evidence

The evidence grade was assessed using the Grading of Recommendations, Assessment, Development and Evaluation (GRADE) system, categorized as “high,” “moderate,” “low,” or “very low.” The GRADE assessment was conducted independently by two researchers.

### 2.7 Statistical analysis

Data analysis was performed using Stata 18.0 statistical software (StataCorp LLC, College Station, TX, USA). Because pain intensity was assessed using VAS scores across the included studies, standardized mean differences (SMDs) with 95% confidence intervals (CIs) were calculated to estimate the pooled effect size. In this meta-analysis, negative SMD values indicated a greater reduction in pain in the exercise therapy group compared with the control group, whereas positive values indicated an effect direction that did not favor exercise therapy.

Considering the expected clinical and methodological heterogeneity among studies, including differences in participant characteristics, intervention protocols, and comparator conditions, a random-effects model was used for pooled analysis. Statistical heterogeneity was assessed using Cochran’s Q test and the I^2^ statistic. An I^2^ value greater than 50% was considered to indicate substantial heterogeneity.

To explore potential sources of heterogeneity, subgroup analysis was conducted according to athlete type. Studies were classified into the racquet sport athlete subgroup when the participants were explicitly described as racquet sport athletes, such as tennis, badminton, or table tennis players. Studies that did not specifically include racquet sport athletes were classified into the non-racquet sport subgroup. The pooled effect size for each subgroup was calculated using the same random-effects model, and differences between subgroups were assessed using the test for subgroup differences.

Sensitivity analysis was performed using a leave-one-out approach, in which each study was sequentially removed and the pooled effect size was recalculated. This analysis was used to examine whether the overall result was driven by any single study and to evaluate the robustness of the pooled estimate.

Publication bias and small-study effects were assessed using funnel plots and Egger’s regression test. Funnel plot asymmetry was visually inspected, and Egger’s test was used as a statistical assessment of small-study effects. A p-value <0.05 was considered statistically significant.

## 3 Results

### 3.1 Literature screening

**FIGURE 1:**
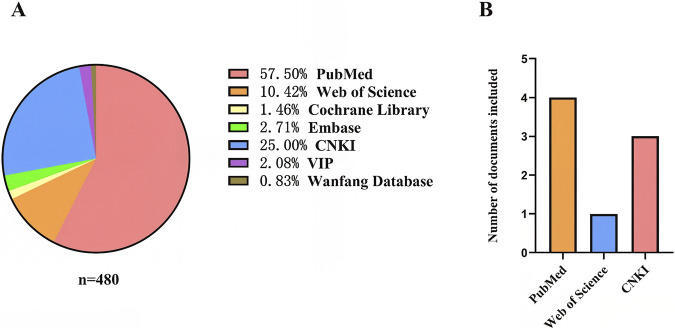
Basic information for literature inclusion. **(A)** Distribution of records retrieved from each database. **(B)** Distribution of included studies by database source.

**FIGURE 2:**
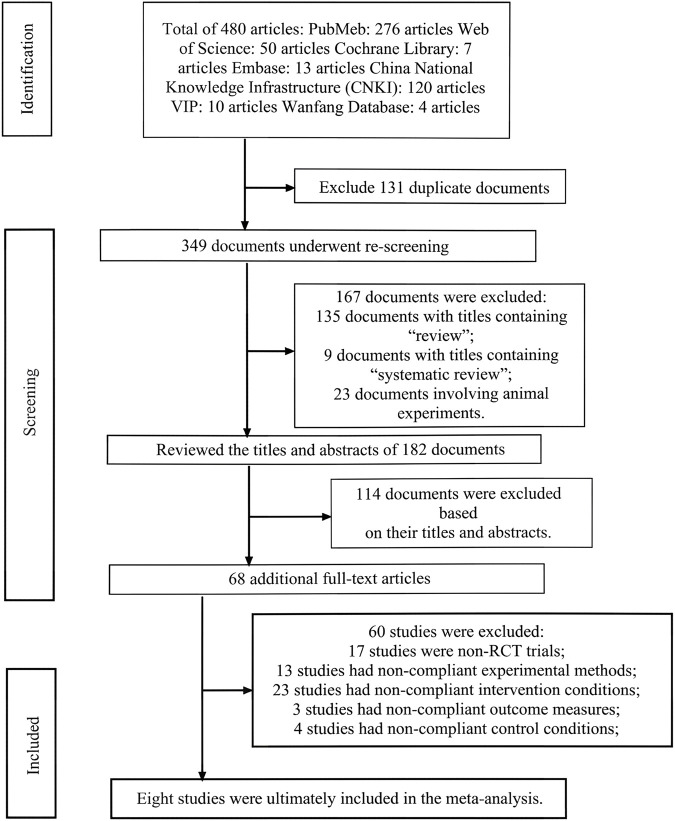
Literature screening process.

This study retrieved 480 articles from seven databases are listed as: PubMed, Web of Science, Cochrane Library, Embase, China National Knowledge Infrastructure (CNKI), Web of Science, and Wanfang. The distribution of retrieved and included records across databases is shown in Figure 1. Based on inclusion and exclusion criteria, eight articles were ultimately included in the meta-analysis. The literature screening process is shown in Figure 2.

### 3.2 Basic characteristics of included literature

**FIGURE 3:**
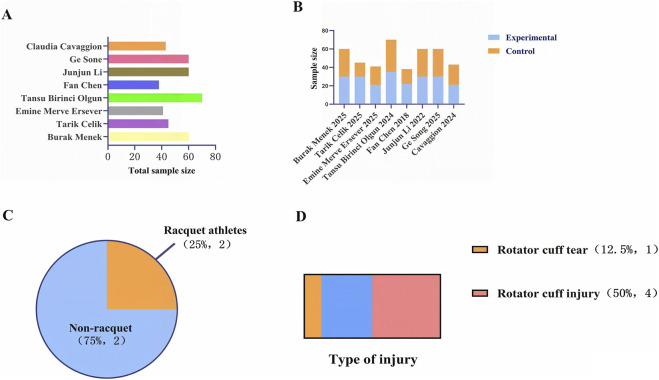
Basic characteristics of the included studies. **(A)** Total sample size of each included study. **(B)** Sample size distribution between experimental and control groups. **(C)** Distribution of racquet and non-racquet athlete populations. **(D)** Distribution of injury types across the included studies.

All eight studies employed a randomized controlled trial design, involving a total of 417 participants, including 218 non-athletes and 199 athletes. The experimental group comprised 219 subjects: 116 non-athletes, 51 racquet athletes, and the remainder non-racquet athletes. The control group included 198 subjects: 102 non-athletes, 50 racquet athletes, and the remainder non-racquet athletes. All enrolled participants presented with shoulder pain related to rotator cuff injuries. Interventions in the experimental group included Mulligan mobilization combined with conventional exercise therapy, steroid injections combined with conventional exercise therapy, conventional exercise plus TENS electrical stimulation, and core stability training. The control group received either physical therapy or surgical treatment. Measured outcomes included VAS scores, range of motion, and Constant-Murley scores. Characteristics of the included studies are summarized in Table 3. The basic characteristics of the included studies are further illustrated in Figure 3. Follow-up information was limited across the included studies. Only one study clearly reported a 6-month follow-up, whereas most studies mainly reported immediate post-intervention outcomes or did not provide sufficient follow-up information for a separate long-term analysis.

**TABLE 3:** Characteristics of included studies.

Author/Publication year	Research design	Sample size (experimental/control)	Is it a racket sport athlete?	Type of injury	Interventions for the experimental group	Control group measures	Outcome indicator
Menek and Menek (2025)	Randomized controlled trial, single-blind	60 (30/30)	No	Rotating sleeve tear	Mulligan mobilization + conventional physical therapy; steroid injection + conventional physical therapy	Standard treatment	VAS; AROM; JPS; DASH
Celik and Menek (2025)	Randomized controlled trial, single-blind	45 (30/15)	No	Supraspinatus tendon injury	Conventional exercise + TENS; maitland technique; mulligan mobilization and warming-up method	Standard treatment	VAS; AROM; JPS; DASH
Ersever and Goktas (2025)	Randomized controlled trial, single-blind	41 (21/20)	Yes	Supraspinatus tendon injury	Core stability training	Standard treatment	VAS; Peak torque of rotator cuff muscles
Birinci Olgun et al. (2024)	Randomized controlled trial, single-blind	70 (35/35)	No	Supraspinatus tendon injury	Clinic-guided training + home-based training	Regular exercise	VAS; AROM
Fan et al. (2018)	Randomized controlled trial, single-blind	38 (22/16)	No	Rotator cuff injury	Conventional exercise therapy	Conventional physical therapy	VAS; ASES; Clinical efficacy
Li (2022)	Randomized controlled trial, single-blind	60 (30/30)	No	Rotator cuff injury	Conventional exercise therapy	Standard treatment	VAS; Constant-murley; AROM
Lafrance et al. (2024)	Randomized controlled trial, single-blind	60 (30/30)	Yes	Rotator cuff injury	Conventional exercise therapy	Standard treatment	VAS; muscle strength
Cavaggion et al. (2024)	Randomized controlled trial, double-blind	43 (21/22)	No	Rotator cuff injury	Conventional exercise therapy	Standard treatment	VAS; AROM

### 3.3 Meta-analysis results

**FIGURE 4:**
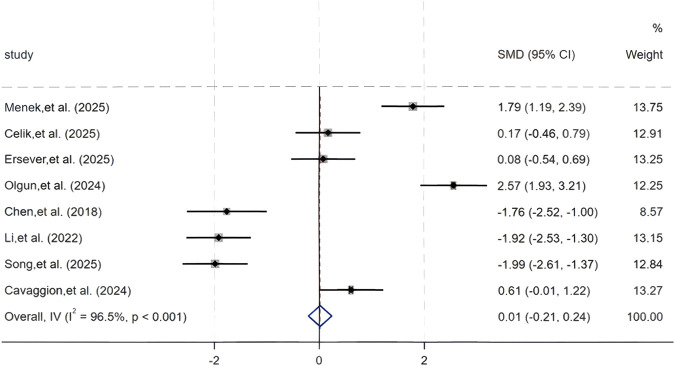
Random-effects forest plot of the overall meta-analysis.

The meta-analysis of eight included studies revealed a pooled standardized mean difference (SMD) of 0.01 (95% CI: 0.21, 0.24) across all studies, with significant heterogeneity (I^2^ = 96.5%, p < 0.001). The wide confidence interval for the overall effect indicates substantial variation among studies, and the effect size being close to zero suggests no significant overall effect. Therefore, the overall pooled result did not support a statistically significant pain-reducing effect of exercise therapy across all included populations. The overall random-effects forest plot is shown in Figure 4.

The subgroup effect sizes were calculated using the same SMD approach and random-effects model as the overall analysis. Studies were grouped according to whether the participants were racquet sport athletes. Subgroup 1 (Racquet Athletes): Studies: Ersever and Goktas (2025) and Ge (2024). The pooled SMD for this subgroup was “-0.94” (95% CI: 1.38, −0.50), indicating a large negative effect for racquet athletes. The overall weight for this subgroup was 26.1%, and heterogeneity was present (I^2^ = 95.4%, p < 0.001), suggesting substantial variation among studies. Subgroup 2 (Non-racket athletes): Studies: Menek and Menek (2025), Celik and Menek (2025), Birinci Dale et al. (2024), Fan et al. (2018), Li (2022), Cavaggion et al. (2024). The pooled SMD for this subgroup was 0.35 (95% CI: 0.09, 0.61), indicating a small positive effect for non-racket athletes. The overall weight for this subgroup was 73.9%, and heterogeneity was also present (I^2^ = 96.7%, p < 0.001), similarly suggesting substantial variation among studies.

**FIGURE 5:**
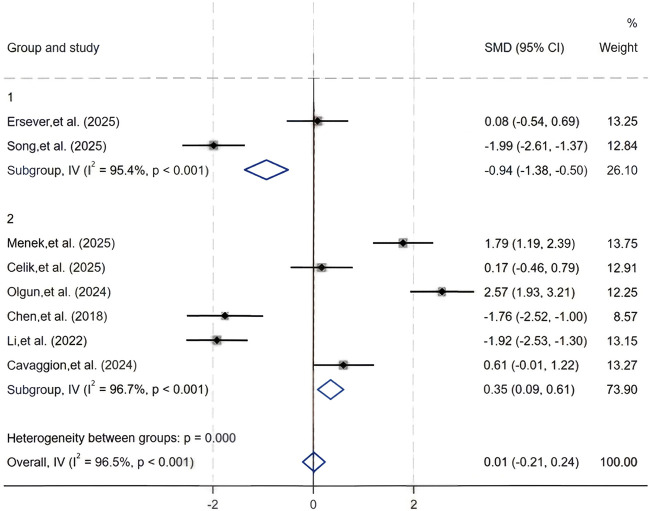
Subgroup analysis forest plot.

Although subgroups differed significantly from each other (p < 0.001), substantial unexplained heterogeneity persisted within both subgroups (I^2^ > 95%), suggesting that athlete type alone does not account for the variability among studies. This indicates that factors beyond athlete type, such as differences in exercise protocols, injury characteristics, intervention duration, comparator interventions, and outcome assessment, may have contributed to the remaining heterogeneity. The subgroup analysis forest plot is shown in Figure 5.

### 3.4 Sensitivity analysis and publication bias risk

**FIGURE 6:**
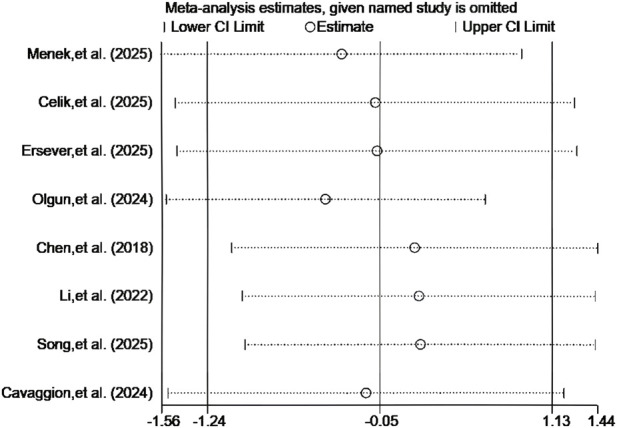
Sensitivity analysis chart.

Sensitivity analysis was conducted using a leave-one-out approach. Each study was sequentially excluded, and the pooled effect size was recalculated to examine whether the overall result was driven by any single study. The results showed that removal of individual studies did not materially change the direction or interpretation of the pooled estimate, suggesting that the overall result was not dependent on a single study. The leave-one-out sensitivity analysis is shown in Figure 6.

Publication bias and small-study effects were assessed using funnel plots and Egger’s regression test. The funnel plot showed visual asymmetry, suggesting the possibility of publication bias or small-study effects. However, because only eight studies were included, the interpretation of funnel plot asymmetry and Egger’s test should be cautious.

### 3.5 Levels of evidence

**FIGURE 7:**
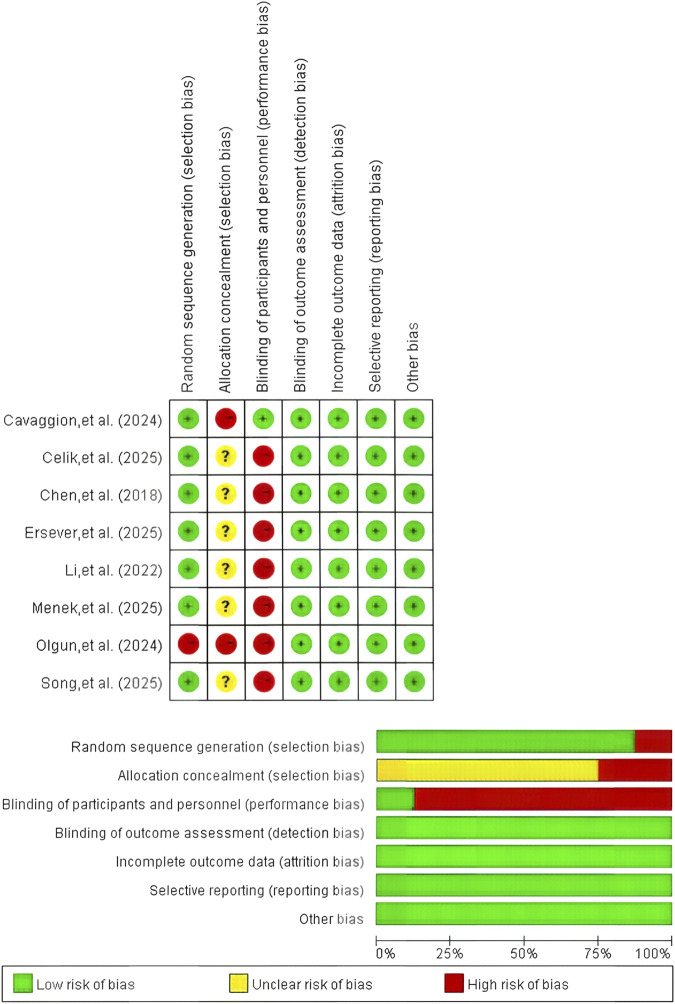
Results of literature bias risk assessment.

The risk-of-bias assessment is presented in Figure 7 and Table 4. Overall, the included studies showed varying levels of methodological quality, with several studies judged as having some concerns or a moderate-to-high risk of bias. No study was excluded from the meta-analysis solely because of its risk-of-bias judgment. However, the presence of methodological limitations was considered when interpreting the pooled estimates and when assessing the certainty of evidence using the GRADE approach.

**TABLE 4:** Assessment of risk of bias and evidence quality.

Research title	Bias risk assessment	Grade
Menek and Menek (2025)	Moderate-to-high risk	Low
Celik and Menek (2025)	Moderate-to-high risk	Low
Ersever and Goktas (2025)	Moderate-to-high risk	Low
Birinci Olgun et al. (2024)	Moderate-to-high risk	Moderate
Fan et al. (2018)	High risk of bias	Low
Li (2022)	High risk of bias	Low
Lafrance et al. (2024)	Moderate-to-high risk	Low
Cavaggion et al. (2024)	Low bias risk	Moderate

Based on the above results, the evidence level from the meta-analysis is considered “moderate.” Despite significant heterogeneity and risk of bias, sensitivity analyses indicate that excluding individual studies does not substantially alter the overall findings, suggesting a degree of robustness in the conclusions.

## 4 Discussion

### 4.1 Summary of evidence

This systematic review and meta-analysis included eight randomized controlled trials involving 417 participants. The overall pooled analysis showed no clear evidence of a difference between exercise therapy and control interventions in reducing shoulder pain related to rotator cuff conditions. The pooled effect size was close to zero, and substantial heterogeneity was observed across studies. Therefore, the overall finding does not support a definitive conclusion that exercise therapy is superior to control interventions for all included populations.

However, subgroup analysis suggested a potential difference according to athlete type. The rationale for interpreting this subgroup separately is that racquet sport athletes are exposed to repeated sport-specific shoulder loading, including rapid arm acceleration, deceleration, and rotational demands. These features may create different rehabilitation priorities compared with non-athlete or general RCRSP populations, particularly with respect to restoring dynamic shoulder stability and sport-specific movement control. In the racquet sport athlete subgroup, exercise therapy was associated with a greater reduction in pain, whereas this pattern was not observed in the non-racquet sport subgroup. Because only a limited number of studies contributed to the racquet sport subgroup and heterogeneity remained high, this finding should be interpreted as exploratory rather than confirmatory.

The high heterogeneity observed in the overall analysis and subgroup analyses may be explained by several clinical and methodological factors. First, the included studies differed substantially in exercise protocols, including core stability training, conventional exercise therapy, Mulligan mobilization combined with exercise, and exercise combined with other physical therapy modalities. These interventions may not produce equivalent effects on pain reduction. Second, participant characteristics varied across studies, including athlete status, sport type, baseline shoulder function, and possible differences in injury severity. In particular, racquet sport athletes are exposed to repetitive overhead or striking movements, which may influence both the nature of shoulder symptoms and the response to exercise-based rehabilitation. Third, differences in intervention dose, including duration, frequency, intensity, progression, and adherence, may have contributed to variability in treatment effects. Fourth, comparator interventions also varied across trials, which may have influenced the estimated between-group effects. Therefore, although the subgroup analysis suggested a potential difference according to athlete type, this factor alone cannot fully explain the observed heterogeneity. The subgroup findings should therefore be interpreted cautiously and regarded as exploratory.

### 4.2 Physiological mechanisms of exercise therapy in improving shoulder pain associated with rotator cuff injuries

Rotator cuff injuries and shoulder pain are common sports injuries in clinical practice, particularly prominent in individuals who frequently engage in upper limb activities or high-intensity exercise (Hoppe et al., 2022). Exercise therapy, particularly physical therapy and functional training, is widely applied to improve symptoms and function in patients with rotator cuff injuries (Lafrance et al., 2024). In the present meta-analysis, the overall pooled effect did not indicate a statistically significant reduction in pain, and heterogeneity across trials was substantial. The following section therefore focuses on plausible physiological pathways that may underpin clinical improvement in selected outcomes or subgroups, and that may contribute to variability in treatment response.

#### 4.2.1 Enhancing shoulder muscle strength and coordination

The rotator cuff muscles play a crucial role in shoulder joint stability (Sangwan et al., 2015; Akhtar et al., 2021). Rotator cuff injuries typically result in weakened strength and impaired function of these muscles (Sangwan et al., 2015). Exercise therapy, particularly strength training targeting the shoulder, alleviates shoulder pain by enhancing the strength and endurance of the rotator cuff muscles, improving dynamic stability of the shoulder joint, restoring rotator cuff function, and reducing joint load (Cho et al., 2024).

#### 4.2.2 Promoting blood circulation and soft tissue repair

Exercise therapy promotes soft tissue repair and enhances nutrient supply by increasing blood flow to the shoulder region (Dominguez-Romero et al., 2021). During the acute phase, moderate exercise reduces muscle adhesions and atrophy, strengthens the repair capacity of muscles and ligaments, improves joint range of motion, and alleviates joint stiffness (Chimenti et al., 2018; Di Rosa et al., 2019; Roldán-Jiménez et al., 2019).

#### 4.2.3 Alleviating inflammatory responses and pain

Exercise therapy effectively reduces local inflammatory responses associated with rotator cuff injuries by promoting muscle and joint mobility. Mild load training stimulates fibroblast and chondrocyte activity, thereby aiding tissue repair and reducing pain occurrence (Dominguez-Romero et al., 2021). Appropriate loading promotes synovial fluid circulation, contributing to reduced local inflammation and pain perception (Di Rosa et al., 2019). Furthermore, exercise therapy enhances pain tolerance and coping strategies through neuromodulatory mechanisms, thereby diminishing the perception of shoulder pain (Chimenti et al., 2018).

#### 4.2.4 Restoring range of motion and function in the shoulder joint

Rotator cuff injuries are often accompanied by reduced shoulder joint range of motion, particularly during movements such as abduction and external rotation. Physical therapy, especially stretching and range-of-motion exercises, can effectively improve shoulder joint mobility, restore normal movement patterns, and reduce functional limitations caused by joint contractures (Gutiérrez-Espinoza et al., 2023; Lee et al., 2023; Lafrance et al., 2024).

#### 4.2.5 Prevention of recurrence and functional rehabilitation

Exercise therapy not only aids in the recovery of shoulder injuries but also reduces the risk of re-injury by strengthening the supporting structures around the shoulder, such as the scapula and shoulder joint ligaments (Liaghat et al., 2023). Functional training and posture correction help athletes return to higher levels of athletic performance while preventing shoulder joint overload and re-injury (Chen et al., 2021; Zawadka et al., 2024).

Consistent with our research, these physiological mechanisms underscore the importance of personalized exercise interventions in rotator cuff injury rehabilitation. Particularly for racquet athletes, targeted training programs can more effectively promote recovery and prevent re-injury, thereby providing crucial reference for future personalized exercise therapy design.

### 4.3 Limitations and clinical implications

Although this study systematically evaluated the effects of exercise therapy on rotator cuff-related shoulder pain, several limitations should be acknowledged. First, the included studies had relatively small sample sizes, and substantial heterogeneity was observed in the pooled analysis, which may limit the generalizability of the findings. Differences in study populations, exercise modalities, intervention intensity, duration, frequency, progression criteria, adherence, comparator interventions, and outcome assessment may have contributed to the high heterogeneity in effect sizes.

Second, although the subgroup analysis suggested a potentially greater pain reduction among racquet sport athletes, this finding was based on a limited number of studies and should be interpreted as exploratory rather than confirmatory. Athlete type alone could not fully explain the between-study variability, as substantial heterogeneity remained within the subgroup analyses. Because of the limited number of included studies, meta-regression or more detailed subgroup analyses based on exercise intensity, intervention duration, injury severity, or baseline function were not feasible. Therefore, the potential sources of heterogeneity discussed above should be interpreted as plausible explanations rather than confirmed determinants. The findings should not be generalized to all individuals with rotator cuff-related shoulder pain. The subgroup findings are more directly relevant to racquet sport athletes, but even in this population, they should be interpreted cautiously because of the limited number of studies and substantial heterogeneity.

Third, the long-term effectiveness of exercise therapy could not be fully evaluated. Follow-up information was limited and inconsistently reported across the included studies. Only one study clearly reported a 6-month follow-up, whereas most studies mainly provided immediate post-intervention outcomes or did not report sufficient follow-up data for a separate pooled analysis. Therefore, the sustainability of pain reduction and functional improvement after exercise therapy remains uncertain. Future randomized controlled trials should include standardized follow-up assessments to determine whether exercise-induced improvements are maintained over time, particularly in racquet sport athletes who may be exposed to recurrent overhead and striking loads.

In addition, potential publication bias may have affected the reliability of the pooled estimates. Although sensitivity analyses indicated that excluding individual studies did not materially change the overall interpretation, unpublished studies and grey literature were not included, which may have increased the possibility of publication bias.

From a clinical perspective, the findings suggest that exercise therapy may have potential value as one component of individualized rehabilitation for rotator cuff-related shoulder pain, particularly among racquet sport athletes (Powell et al., 2024). However, because the overall pooled effect was not statistically significant and substantial heterogeneity was present, exercise therapy should not be interpreted as uniformly effective across all populations based on the current evidence. For racquet sport athletes, rehabilitation professionals should consider not only symptom reduction, but also shoulder strength, scapular control, range of motion, neuromuscular coordination, load tolerance, and gradual return-to-sport progression (Schwank et al., 2022). Practical recommendations may include progressive rotator cuff and scapular strengthening, controlled range-of-motion exercises, core and trunk stability training, movement correction, and gradual exposure to sport-specific hitting or overhead tasks (Brumitt and Dale, 2009). These recommendations should be applied cautiously and adjusted according to symptom severity, training level, pain response, and individual recovery status.

Future studies should use larger sample sizes, standardized intervention protocols, clearly defined exercise intensity and progression criteria, and longer follow-up periods. Further trials are also needed to compare different exercise modalities and to examine whether exercise therapy provides additional benefits compared with other conservative or combined rehabilitation strategies. In racquet sport athletes, future research should also evaluate whether sport-specific rehabilitation programs can produce sustained improvements in pain, shoulder function, load tolerance, and return-to-sport outcomes.

## 5 Conclusion

This systematic review and meta-analysis found no clear evidence of an overall difference between exercise therapy and control interventions in reducing pain among individuals with rotator cuff-related shoulder pain. Although subgroup analysis suggested that exercise therapy may be associated with greater pain reduction among racquet sport athletes, this finding should be interpreted cautiously because of the limited number of studies, substantial heterogeneity, and potential publication bias. Current evidence does not support an overgeneralized conclusion that exercise therapy is uniformly effective across all RCRSP populations. The subgroup finding may be more relevant to racquet sport athletes, but it should be interpreted cautiously because of the limited number of studies, substantial heterogeneity, and potential publication bias.

## Data Availability

The original contributions presented in the study are included in the article/Supplementary material. Further inquiries can be directed to the corresponding author.
